# Supplementary material for: Growth Inhibition by Amino Acids in Saccharomyces cerevisiae
Source: Microorganisms. 2020 Dec 22;9(1):7. doi: 10.3390/microorganisms9010007 (PMC7822121; doi:10.3390/microorganisms9010007)
Supplement: Supplementary file 1 [file microorganisms-09-00007-s001.pdf]

microorganisms

MDPI

Supplementary Information

Article

Growth Inhibition by Amino Acids in *Saccharomyces Cerevisiae*

Stephanie J. Ruiz [1], Joury S. van 't Klooster [1], Frans Bianchi [1] and Bert Poolman [1,*]

[1] Department of Biochemistry, Groningen Biomolecular Sciences and Biotechnology Institute, University of Groningen, Nijenborgh 4, 9747 AG Groningen, The Netherlands; s.j.ruiz@outlook.com (S.J.R.); f.bianchi@rug.nl (F.B.)

* Correspondence: b.poolman@rug.nl; Tel.: +31-50-363-4190; Fax: +31-50-363-4165

Keywords: amino acid transport, amino acid toxicity, growth inhibition, *Saccharomyces cerevisiae*

Supplementary Information: Sequence of fluorescent protein that was engineered at the C-terminus of the amino acid transporters, Figure S1 – S6

The C-terminal tag, whose full amino acid sequence is shown below, contains a TEV protease recognition site (underlined) followed by the fluorescent protein YPet (in bold) and an eight-residue His epitope (in italics). The residue pairs “GG” and “EL” are a short linker and a cloning artifact, respectively.

GGENLYFQG**SKGEELFTGVVPILVELDGDVNGHKFSVSGEGEGDATYGKLTLKLLCTTGKLPVPWPTLVTTLG YGVQCFARYPDHMKQHDFFKSAMPEGYVQERTIFFKDDGNYKTRAEVKFEGDTLVNRIELKGIDFKEDGNIL GHKLEYNYNSHNVYITADKQKNGIKANFKIRHNIEDGGVQLADHYQQNTPIGDGPVLLPDNHYLSYQSALFKD PNEKRDHMVLLEFLTAAGITEGMNELYK**EL*HHHHHHHH*

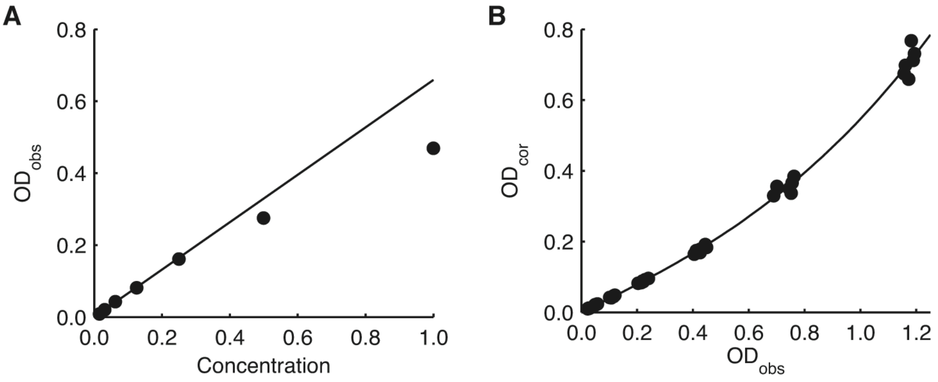

Figure S1. OD_{600} correction for linearity at high cell density. (**A**) OD_{600} measurements after blank subtraction (OD_{obs}) from a sample dilution series. Values judged to be in a linear range were fit with a straight line to generate a corrected OD (OD_{cor}) for each concentration. (b) OD_{obs} and OD_{cor} values for six dilution series from three independent experiments were fit with a cubic polynomial ($OD_{cor} = 0.319 \times OD_{obs}^3 + 0.089 \times OD_{obs}^2 + 0.959 \times OD_{obs}$).

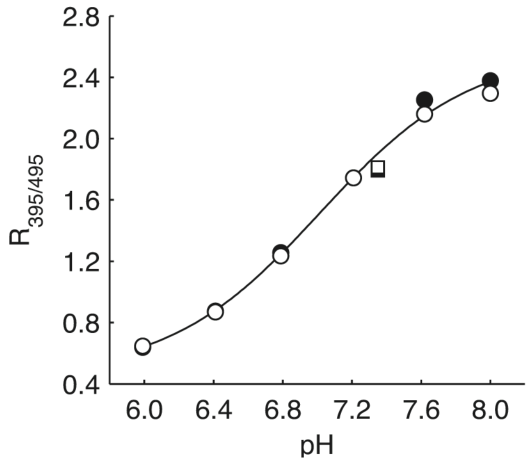

Figure S2. Calibration of pHluorin fluorescence. Cells expressing pHluorin (open symbols) or pHluorin and Lyp1 (closed symbols) were semi-permeabilized using digitonin and diluted into 100 mM KPi (circles) or PBS (squares) buffers. Emission intensity at 508 nm was measured using excitation at 395 and 475 nm, and the ratio ($R_{395/495}$) plotted against buffer pH. The KPi data were fit with a modified Henderson-Hasselbalch equation: pH = pK_a’ + $\log_{10}$ (($R_{395/495} - R_{min}$)/(R_{max} - $R_{395/495}$)), where pK_a’ = -$\log_{10}$ of the apparent acid dissociation constant, and R_{min} and R_{max} = the $R_{395/495}$ at extreme low and high pH, respectively.

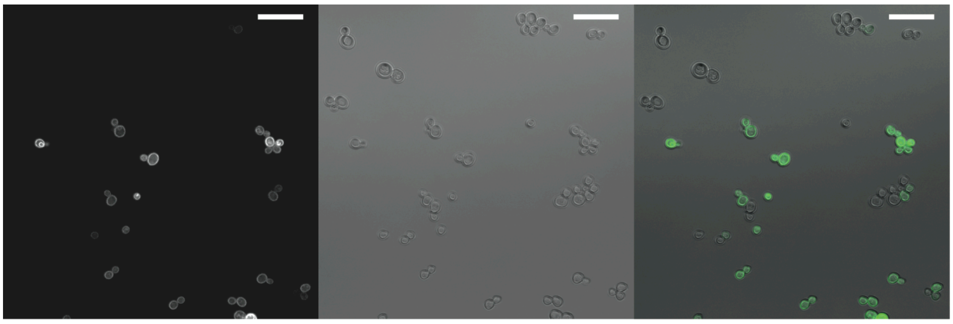

Figure S3. Confocal microscopy images showing the variation in fluorescent signal between cells in the same culture. The strain pictured is expressing Tat2YPet, although similar results were seen for all the overexpression strains. Left = fluorescence, center = brightfield, right = overlay.

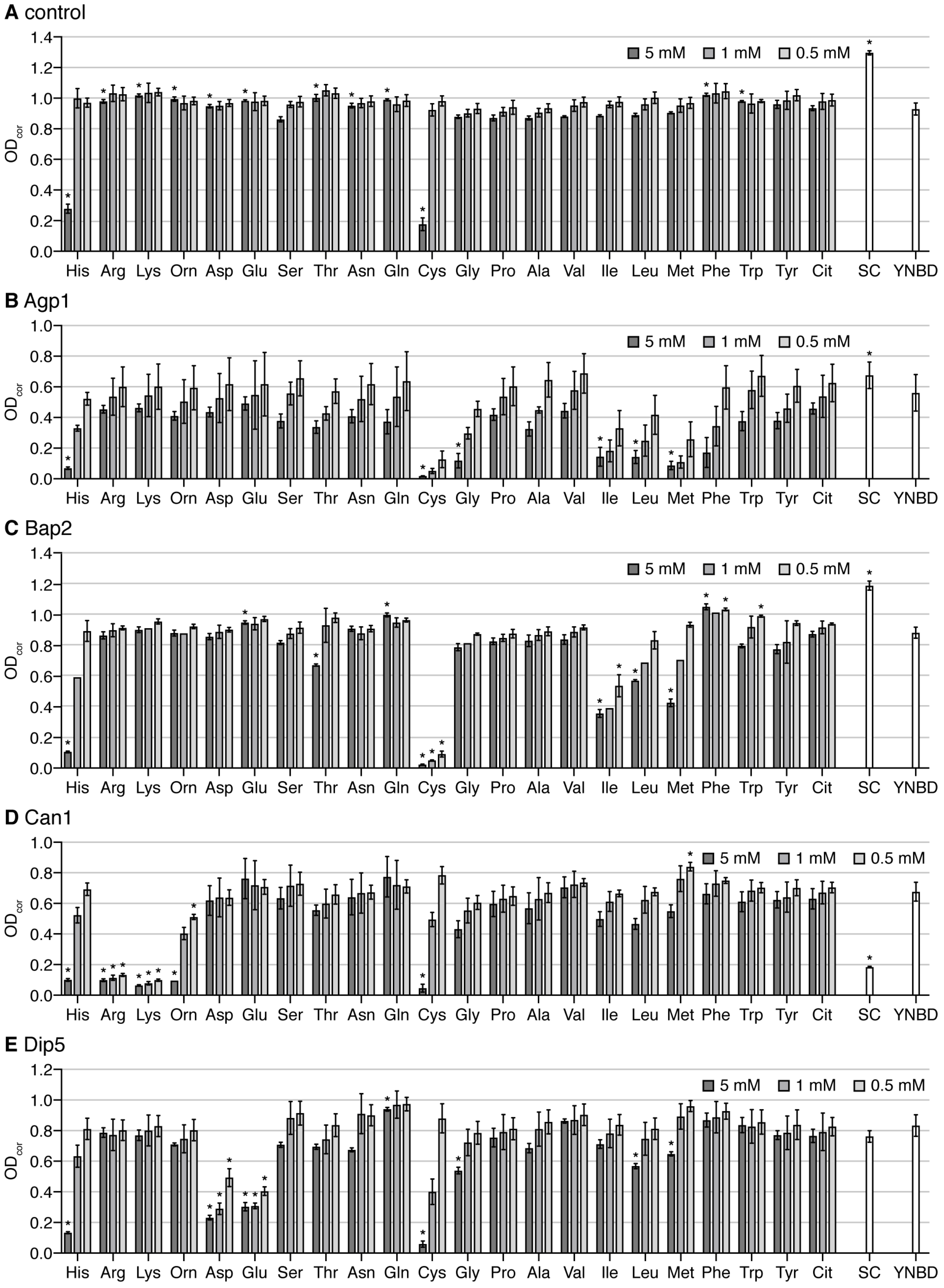
A control
B Agp1
C Bap2
D Can1
E Dip5
ODcor
5 mM
1 mM
0.5 mM
His Arg Lys Orn Asp Glu Ser Thr Asn Gln Cys Gly Pro Ala Val Ile Leu Met Phe Trp Tyr Cit SC YNBD

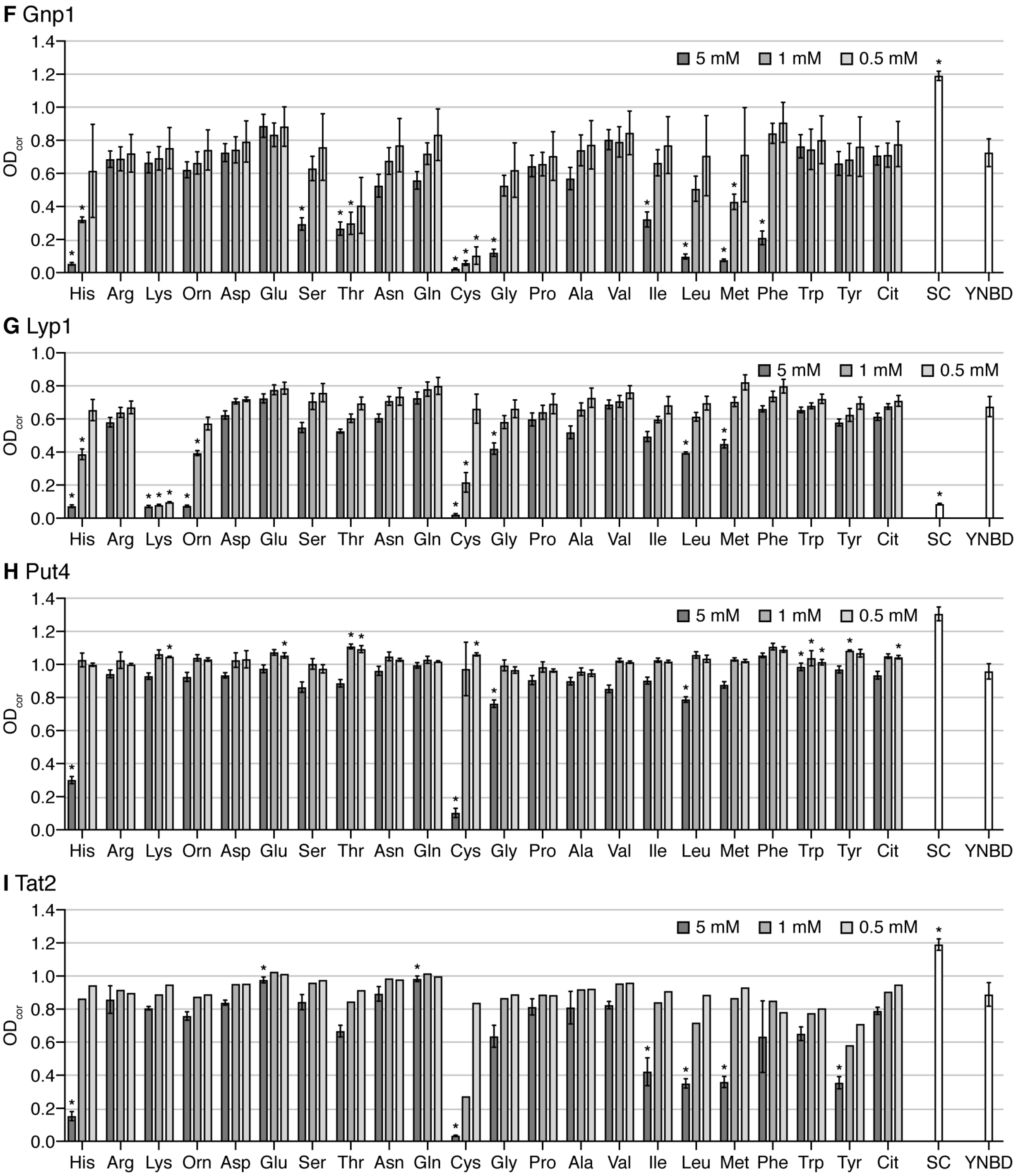

Figure S4. Raw data from experiments presented in Figure 2, showing the effect of amino acids on the growth of strains overexpressing amino acid transporters. BY4709 carrying the empty plasmid pRSII426 was used as a control. All proteins were expressed from the constitutive ADH1 promoter with a C-terminal YPet tag. The proteinogenic amino acids are represented by their three letter codes (see Table 4). Orn = ornithine, Cit = citrulline, SC = synthetic complete media minus uracil, YNBD = minimal medium without amino acids. Amino acids were at final concentrations of 0.5, 1, or 5 mM. OD_{cor} is the measured OD_{600} after 24 h of growth, blank subtracted and corrected for linearity at high cell densities (see Materials and methods). Values shown are the mean ± standard deviation (n = 3 for amino acids and SC, n = 9 for YPD). Due to technical error some results had to be discarded and therefore n = 2 for Bap2 1 mM Gly/His/Ile/Leu/Lys/Met/Orn/Phe, and also for all Tat2 1 mM and 0.5 mM conditions (no error bars are given for these). Although this figure shows the mean of all replicates, the normalized heatmaps in Figure 2 and the statistical analyses were generated by comparing specific pairs of growth values (e.g. YNBD vs YNBD + Cit for samples from the same microplate, inoculated from the same preculture). Asterisks indicate $p < 0.05$ when compared to growth in YNBD (*t*-test).

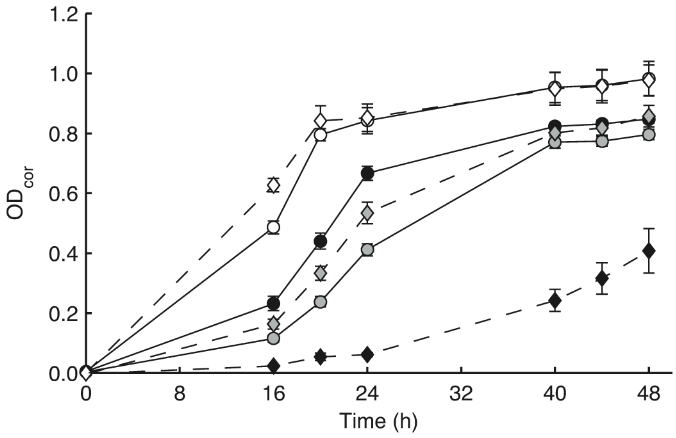

Figure S5. Growth curves from the same experiment shown in Figure 4. Shown here are cultures grown with no external amino acids (circles, solid lines) or with 500 µM arginine (diamonds, dashed lines). Strains without the arginine sensitivity phenotype experience a growth advantage in the presence of external arginine. At t = 24 h, the time-point shown in Figure 4, this advantage is only apparent for the slower growing Lyp1 overexpression strain. Open symbols = BY4709 carrying the empty plasmid pRSII426, closed symbols = cells overexpressing Can1, shaded symbols = cells overexpressing Lyp1. All values shown are the mean of biological triplicates. Error bars represent standard deviation and in some cases are obscured by the data point.

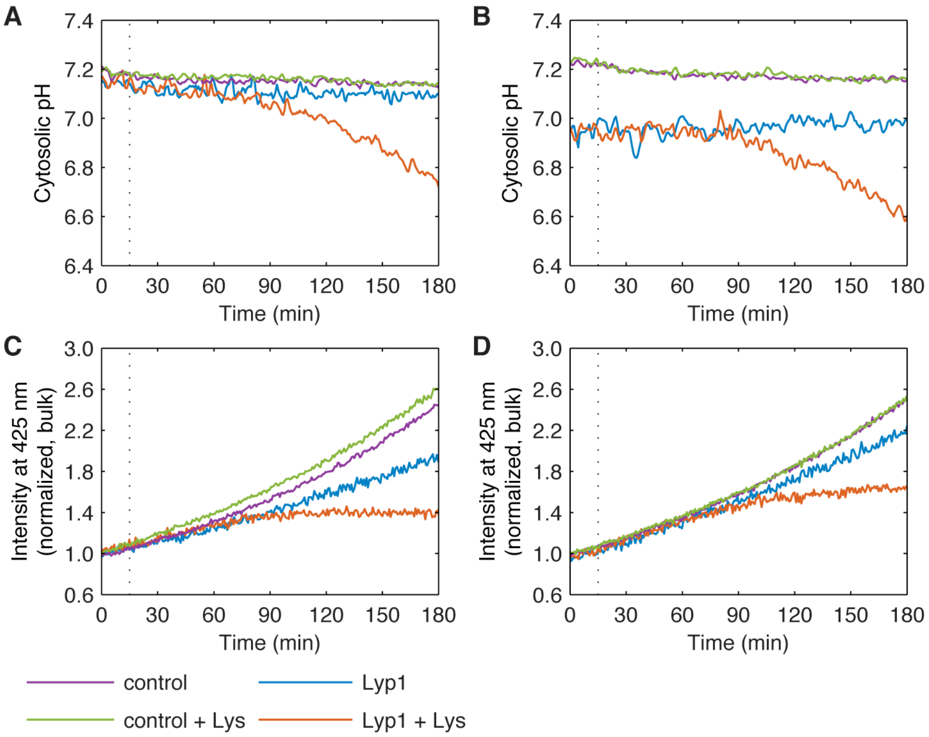

Figure S6. Data from the two experiments used to generate Figure 5. Data in (A) & (C) and (B) & (D) were collected on two separate days, using independent cultures. The pH values were calculated from a single calibration curve shown in Figure S5.
